# Local newspapers and coronavirus: conceptualising connections, comparisons and cures

**DOI:** 10.1177/1329878X20956455

**Published:** 2021-02

**Authors:** Kristy Hess, Lisa Jane Waller

**Affiliations:** Deakin University, Australia; RMIT University, Australia

**Keywords:** coronavirus, local media, media policy, news business models, newspapers, social sphere

## Abstract

Within weeks of the nation-wide COVID-19 shutdown, more than 200 regional and community newspapers across Australia announced they could no longer keep their presses running due to the unprecedented crisis. A drain in advertising spend, a broken business model and the refusal of digital behemoths to pay for content were blamed for their collapse, ironically as audiences’ demand for credible news and information soared across the globe. There is no doubt the COVID-19 crisis has widened existing, deep cracks in the news media industry. In response this article sets out to explore possible solutions and strategies for local newspapers in the post-pandemic media landscape. We take an analogical approach to argue some of the issues that emerged during COVID-19 and strategies used to fight the global health pandemic also present valuable lessons for the preservation of public interest journalism and news at the local level. We conceptualise five coronavirus-related themes that resonate with a much-needed innovations agenda for local newspapers in Australia: (1) support for essential services, (2) warnings of complacency against an evolving biological threat, (3) appreciating the power of the social (4) coordinated government/policy responses and (5) ‘we are all in this together’.

## Introduction

In April 2020, at the height of the COVID-19 lockdown in Australia, University of Sydney emeritus professor of public health Simon Chapman wrote a piece of media commentary highlighting the power of analogy in countering cognitive dissonance, a term that describes the ability of people to sustain particular beliefs or behaviours in the face of overwhelming evidence that could shatter their views. He argued that during his work as a public health advocate, analogy had proved to be a useful tool for dismantling steadfast beliefs in the interests of positive social change. The story included a graphic of the following analogy to describe sections of Australian society’s eagerness to ease COVID-19 social distancing restrictions:

The curve is flattening, we can start lifting social restrictions now = the parachute has slowed our rate of descent, we can take it off now? (Chapman, 2020, n.p)

An area of society that has taken a major nosedive during the global pandemic, and according to a chorus of media reports requires a parachute for safe landing, is the news industry itself (see Meade, 2020). Despite a sharp rise in demand for reliable and relevant news and information due to the virus (Park et al., 2020), there were more than 200 regional newspaper closures in Australia and/or temporary suspensions of the printed publications (Public Interest Journalism Initiative, 2020) between January and June 2020. Some of these publications including the *Yarram Standard* in Victoria, which first rolled off the press more than 140 years ago, survived the Spanish flu of 1918-19 and flourished for decades afterwards. However, the Australian media’s underlying health issues meant these newspapers had little or no immunity when the acute economic downturn that accompanied COVID-19 infected their life blood.

Newspapers’ deteriorating state of wellbeing–with symptoms including shrinking readerships and weakening advertising revenue–has been charted by media watchers dating back to the advent of radio and then television (Temple, 2017). The decline of print was declared to have entered a more acute phase in the early 2000s when Web 2.0 revolutionised the way digital information is created, shared, stored, distributed, and manipulated. The enduring discourses of ‘crisis’ and ‘digital disruption’ that emerged have contributed not only to how journalism is perceived, but also have shaped its reality (Zelizer, 2015). For example, both industry and academics have been in a perpetual state of anxiety over the ‘future of journalism’, with countless conferences, summits, roundtables, industry and government reports, policy overhauls and literally thousands of academic papers and books dedicated to the topic. There have also been many experiments in the search for a ‘cure’ – from enterprises including AOL’s failed hyperlocal experiment Patch, to local news outlets recruiting ‘citizen journalists’ to provide content or seeking philanthropic partnerships to underwrite public interest journalism (Hess and Waller, 2019).

One of the most remarkable aspects of COVID-19 has arguably been how rapidly it overwhelmed the dominant discourses of ‘crisis’ and ‘digital disruption’ in journalism studies and practice. This special issue of *Media International* is one of many such themed editions throughout the world with a strong focus on viewing journalism through the lens of the pandemic. As researchers working on a project that will gauge the civic value of Australia’s country press and develop an innovation model for the sector,^1^ we have made the case for an approach to media innovation that does not simply reinforce the ‘crisis’ discourse (Hess and Waller, 2020) or pin small newspaper closures like butterflies to a Google map of pandemic despair (see https://piji.com.au).

Instead, we set out to learn from the unexpected within scholarship, industry and policy arenas and how responses to the current health crisis might inform new directions for achieving and maintaining a safe and sustainable local newspaper sector. In the sections that follow we will first provide some context around the state of local newspapers prior to the onset of COVID-19. We look to interdisciplinary scholarship on analogy to argue the pandemic can play a heuristic role in developing fresh approaches related to local journalism practice, policy and theory. Examining some of the *strategies* and *tactics*^2^ used to tackle COVID-19 through the conceptual framework of five pandemic-related themes presents valuable lessons and synergies in the fight to preserve the health of public interest journalism in Australia.

### Vulnerable newspaper communities and the fight to survive

When COVID-19 virus hit Australia’s shores, it circulated predominantly in the nation's major metropolitan areas, leaving rural areas largely unscathed. However, long-serving rural newspapers were arguably hardest hit by the sudden decline in media advertising revenue (see e.g. Meade, 2020). Dozens of vulnerable small independent and Australian Community Media publications had little choice but to temporarily freeze their operations and put their cadet journalists on the Government’s JobKeeper scheme. The nation’s media juggernaut News Corporation soon followed with the announcement it would close more than 100 suburban and country newspapers, or shift them to a digital-only format (Mason, 2020).

Our research, along with studies produced internationally, highlights that local news matters to audiences and a healthy democracy (see Ali et al., 2018; Hess and Waller, 2017). The vast geographic distances between Australia’s towns and cities, however, generated challenges and opportunities for local media before COVID-19 (Hess and Waller, 2020). Issues facing the sector range from Internet connectivity (Freeman, 2015) to the rise of news gaps in remote areas, or on the periphery of established news circulation or broadcast licencing areas. The overarching policy challenge for Australia’s federal government has been to how best to serve the news and information needs of all Australians, especially when it is well established that the availability of local news and information is considered vital for enhancing civic life and social capital (Richards, 2014).

It is important to note that Australia’s local newspaper sector has been a tale of mixed fortunes since the rise of digital platforms and social media giants like Facebook more than a decade ago. While many newspapers are in decline, some have been in a holding pattern with circulation and revenue across print and digital platforms (see https://countrypressaustralia.com.au). Increasingly Australia is also witnessing an increase in the number of new start-up ventures emerging to fill news gaps or serve communities (see https://papernews.com.au/). Most experts agree, nonetheless, that a comprehensive reassessment is needed, along with innovative strategies for the long-term sustainability of the sector (see Australian Consumer and Competition Commission (ACCC), 2018). There have been two national media policy developments in recent years that have sought to define the problems besetting the country newspaper sector and generate solutions and these have evolved or been refined during COVID-19. The first was the Federal Government’s innovation fund for rural and regional publishers that followed a series of Senate inquiries into public broadcasting and public interest journalism between 2015-2018 (Australian Government, 2018b). The $48 million, three-year Regional and Small Publishers Innovation Fund (Australian Government, 2018a) was developed to support ‘. . . the continuation, development, growth and innovation of Australian journalism that investigates and explains public policy and issues of public significance, engages citizens in public debate, and informs democratic decision-making’ (Australian Government, 2018a). It adopted a technological determinist approach to innovation (Zelizer, 2015, 2019) by providing support for equipment, software, training and some funds to employ cadet journalists. During the pandemic, the fund was partially repackaged into a $50 million Public Interest News Gathering Programme open to most news providers in the country beyond the rural and regional and a $5 million Regional and Small Publishers Innovation Fund to target small newspaper proprietors (see ACMA, 2020).

The second development was the 2018 ACCC inquiry into the impact of digital platforms, including Google and Facebook, on Australian news and society (Australian Government, 2018b). The biggest proposed shake-up of the sector–to be discussed later in this essay–came in May, 2020 when the Federal Government announced plans to force Facebook and Google to share its advertising revenue to create a more level playing field with news providers (Taylor, 2020). The ACCC has since been given responsibility for developing a mandatory bargaining code to structure the sharing of advertising revenue among Australia’s media players. Other actors have also had influence on supporting local journalism at the local level, from philanthropists to academics and union bodies, which we will also return to soon.

### The imaginative, sensibility and power dimensions of analogy

Our approach to exploring the relationship between COVID-19 and the future of local newspapers in Australia is a novel one because we extend upon a framework for analogising in a way that appreciates its imaginative, sensemaking and sensibility dimensions (see Schwartz Plaschg, 2018). Importantly, this perspective acknowledges the argumentative and persuasive power of analogy. In our broader scholarship, we are also interested in teasing out how analogies can be exploited and strategized by those in positions of influence and power, including academic researchers. In the sections that follow analogy is employed as a heuristic device to aid discovery, innovation, exploration and clarification (Bartha, 2019). In other words, it provides a conceptual shortcut in an attempt to develop, and begin to explore, a new set of arguments about the complex problems and issues facing local journalism. Such analogising, or analogical reasoning, has resulted in major breakthroughs in other disciplines from philosophy to science, law, mathematics, organisation studies, design, cognitive psychology, bio sciences, bioethics, business and creative entrepreneurship (see Steadman, 2008).

In this case, we draw inspiration from the imaginative and sensibility dimensions of analogic reasoning as conceptualised by Claudia Schwartz Plaschg (2018) to help envisage or reimagine how existing structures and thinking around local newspapers might be renewed, reformed or rejected. This approach acknowledges the imaginative function of analogy as the ‘power of the possible’ (Ricoeur, 1992) that can assist in exploring the potentialities of reality, while acknowledging that the ‘imaginative’ needs to be balanced with examining those in positions of privilege and power who utilise analogies for their advantage. Persuasive analogies are often built from a common knowledge source with which both speakers and audience are familiar. Schwartz Plaschg (2018) reminds us that such analogies enact power by evoking widely shared interpretations in their audiences. Analogising therefore calls for researchers, policymakers and industry to practice reflexivity; that is, to deliberately and consciously examine our own motives, practices and positions of power in using analogies to influence society.

We acknowledge that elsewhere we have drawn analogies between hyperlocal journalism ventures and organic mushrooms and asked other journalism academics and policymakers to consider Australia’s three-tiered political system in terms of layers of a wedding cake to foreground more complex theoretical ideas (Hess and Waller, 2016, 2017). By so doing, we have influenced academic understanding and further research on local journalism. We are not alone in using analogy to define the terms of discussion in our field. Journalism as both culture and practice has been analogised in terms of ecosystems, woven fabric, pens and swords. However, the use of the analogy in journalism studies is often adopted with little reflexivity or thought about its purpose or value as a specific research approach, rather adopted instinctively as a strategy to help unpack complex theoretical ideas.

This article contributes to our wider project on the future of country newspapers in Australia and in being reflexive, we appreciate that we hold an immediate bias in that our research aims to not only investigate, but also develop innovative approaches that can help to ensure the sustainability of these local news outlets. In doing so, we openly declare we have an agenda and therefore analogies are a *tactic* we use to discuss the unexpected in our attempt to shift away from the dominant techno-determinist and ‘public interest journalism’ discourses that currently dominate industry, public and policy understanding of the issues at stake (Hess and Waller, 2020). We are eager to move beyond discussions of ‘crisis in the age of COVID-19’ by employing analogy to develop, and begin to explore, a new set of arguments generated by how other powerful institutions have responded to the pandemic and the key challenges and issues that arose in debate about the news. We take five analogies associated with the pandemic to address the future of country newspapers, drawing on media coverage of key themes that emerged during the height of the crisis in March to June 2020. They are: (1) support for essential services, (2) warnings of complacency against an evolving biological threat, (3) appreciating the power of the social (4) the need for coordinated government/policy responses and 4: We are all in this together.

### Support for essential services

‘Essential service’ was arguably one of the most commonly used and somewhat confusing keywords to emerge from Australian leaders’ daily press conferences and news media coverage. It took on new significance and was repurposed in particular ways. For example, there was public debate over whether hairdressing and personal training were ‘essential services’, while according to Prime Minister Scott Morrison everyone with a job was performing an essential service (No Author, 2020a). During the pandemic, local newsrooms shut down throughout the world. In Australia, they were eligible for the JobKeeper scheme, but it did not stop hundreds of newspapers (largely News Corp and ACM) from temporarily or permanently suspending their printed publications and shifting to digital, bringing local journalism into national and international conversations that defined journalism as an ‘essential service’ (Gorman, 2020).

There is no doubt that country newspapers have been undergoing digital transformations that affect many aspects of their operations. Here we argue that it is important to look beyond the role of journalism itself as an essential service to the communities and broader society it serves to also consider the ‘essential service’ role that the printed newspaper plays in rural and regional Australia within a digital world. This factor sets country mastheads apart from many of their national and metropolitan cousins, for whom print is no longer the main game. Journalism scholars, immersed in the increasing scholarship around platformatization, could be forgiven for missing this crucial point as print is fast becoming redundant in much international research on media innovation (Hess and Waller, 2020). Positioning the printed newspaper as an essential service in 2020 is context specific and in response to existing demographics and media-related practices.

Elsewhere we have emphasised the continuing importance of the printed newspaper in times of crisis (Freeman et al., 2018). Online content about COVID-19 is not a service for those with poor connectivity or limited socio-economic circumstances (such as constraints related to age, income and literacy levels) in rural and regional areas. In these places, local journalists are best placed to provide accessible news and information and contextualise global and national stories for their audiences; and significantly, to report how a crisis like COVID-19 is affecting their own institutions, businesses and people. It provides a record of history in these times as well as in everyday life, especially when digital archiving of rural and regional publications are inconsistent and often incomplete in libraries and across archiving platforms. Connectivity challenges also inhibit alternative sources like hyperlocal community media (that are often dependent on more cost effective blog platforms) from reliably enhancing local media responses to crisis.

There is also limited scholarly research on the news needs of ageing residents in rural and regional Australia. While there was intense focus on the health and wellbeing of elderly people during COVID-19 (with early research pointing to the ageing as most susceptible to the virus), the wellbeing of older residents is lacking in academic discussions about the future of local newspapers. An industry survey of more than 470,000 elderly residents by National Seniors Australia (2019) revealed diversity in terms of ability, comfort and attitudes to overall digital literacy and engagement in an online world. When it comes to news use, however, several studies show older residents continue to have an affinity with traditional news sources, especially local press (Barnes, 2015; Nossek et al., 2015; Park et al., 2020). Barnes (2015), for example, argues older Australians continue to rely on ‘traditional’ media for their news and informational needs more than any other information source. The reasons for this include a lack of digital literacy, as well as other exclusion issues including access and cost. She also highlighted other barriers for engagement such as the design and usability of news websites to ensure these are perceived as relevant and usable for older readers.

COVID-19 has sharpened public awareness that many once taken-for-granted services are ‘essential’, with supermarket workers and cleaners celebrated as national heroes alongside medical workers. Recognition of news media as an essential service has provided a strong rationale for policy interventions to support journalism. At the rural and regional level, the discussion has underlined the importance of the printed product in serving the public interest function of serving all sectors of the community which warrants its place as an essential service as an issue of equity during this period of digital transformation.

### Warnings of complacency against an evolving biological threat

Nations throughout the world have scrambled to gather the resources necessary to respond to COVID-19 and the race to find vaccines and treatments is difficult. Critics say there should have been better preparations and resources in place as scientists have been warning for years about an imminent biological threat (Dulaney, 2020). There are parallels here with society’s complacency about the failing health of small newspapers, and then panic, to rescue them amid the pandemic.

Sociologist Robert Park – a founder of local journalism studies–drew an analogy between newspapers and evolutionary adaptation in the 1920s by comparing their evolving norms to the features of a ‘surviving species’ (Park, 1923, see Nadler, 2019 for full discussion). He contended the history of the newspaper ‘is the history of the surviving species. It is an account of the conditions under which the existing newspaper has grown up and taken form” (Park, 1923: np). In this article we compare social media giants such as Google and Facebook to a bacterial infection in Australia’s mediated democracy that are proving a threat to the species’ survival. Policymakers, industry and academics (including ourselves) have all argued during the pandemic that local mastheads are a threatened species that warrants protection as they continue to evolve in the digital era.

Bacteria is a useful analogy for social media because it can be understood to be both dangerous and beneficial and can cause infection if left untreated. The benefit of social media platforms such as Twitter and Facebook to advanced liberal democracies is the subject of much scholarship and debate. On the one hand, societal and political significance of social media, bloggers and the Internet more broadly has been celebrated as part of a new Fifth Estate (Dutton and Dubois, 2015; Jerico, 2012). The bacteria is perceived in some quarters, however, to be dangerous to democracy with Google and Facebook in particular considered to be no ‘friend’ of public interest journalism. Government inquiries across the globe into the platform’s handling of fake accounts, advertising, privacy, and hate speech has revealed how social media sites pillage and plunder the information highway once dominated by traditional mainstream outlets (ACCC, 2018; Halpern, 2019). Analogising tech giants as bacteria shifts the discussion from the rise of digital technology to one that considers the makeup and environmental conditions in which it flourishes or damages healthy mediated discussion. Treatments and vaccines (such as an ACCC bargaining code discussed earlier) are being developed to ensure that the healthy bacteria social media does contribute to public debate in the form of free speech and social connection is not overrun by the kind that is toxic (think misinformation, trolls, fake news) (Tandoc et al., 2020).

We should not, however, overlook the role that news media and journalism studies scholars play in feeding the bacteria that Facebook has become. If we wind the clock back just a decade, it’s easy to forget that the relationship between journalism and Facebook wasn’t all bad. While the shift to digital classifieds had started eating into traditional media advertising during the early 2000s, mainstream news media was still arguably in control of the public conversation. Back then, scholars and industry were fascinated by Facebook as a ‘new play thing’ in the digital sphere. Both journalists and Facebook positioned the platform as a relatively harmless phenomena, given its links to notions of ‘friending’, ‘liking’, and building ‘community’. Journalists began using Facebook to share and promote stories, to find news sources, and to move audiences connected to shared geographical spaces into easily accessible online communities. Almost all local news outlets across Australia now have Facebook pages to promote content. The idea that Facebook could, however, exploit the symbolic power of news media and set journalism aside as a social force just wasn’t a key part of the initial discussion (Gutsche and Hess, 2019).

### Appreciating the power of the social

The directive for entire populations to stay confined to their homes for weeks on end during the COVID-19 pandemic highlights the basic–yet often taken for granted–importance of social interaction and connection. The early collapse of event businesses, cafes and restaurants quickly demonstrated how economies are built around our very desire to be social and the freedom to engage in ritualistic practices such as weddings, celebrations and funerals. Global tech companies with the software and capabilities to connect people socially in a virtual world have been the big winners–sites like Zoom, House Party and live streaming companies, have enjoyed early spikes in usability and profits (No Author, 2020b). The ability to capitalise on our desire for social connection in a mediated world is perhaps best illustrated by the rise of Facebook. At a time when local news media profits have slumped, the company recorded a 10 per cent increase for the first quarter of 2020 and a rise in the number of users (Horwitz, 2020) – a point we shall return to soon.

The focus on the social dimension of our everyday lives provides a powerful reminder of its importance to the future of local newspapers. Yet far too often the ‘social’ dimensions of journalism have been set apart or ‘distanced’ from its revered Fourth Estate function where journalists serve a public sphere and hold power to account. By social we mean the information relevant to the realm of our everyday lives, which helps us make sense of who we are as individuals and collectives (Hess and Gutsche, 2018). Such information may be referred to as stories recognising the success, milestones, tragedy, despair, honour of individuals (human interest stories), solutions journalism that brings people together to advocate social change, curated and contextualised weather and traffic and transport reports/incidents, reporting and synthesis of events, obituaries, feature articles, travel and lifestyle content. Instead, within journalism practice, the social has become synonymous with social media and social networking, as if sites such as Facebook are now the legitimate sphere for our everyday social needs. Terms such as social journalism (Hermida, 2012), social news (Goode, 2009), and the sociability of news (Phillips, 2012) have been coined to explore how social networking is shaping journalism, from its celebrated Fifth Estate function (Jerico, 2012) to audience and journalistic engagement and participation.

There is a need to engage with innovative strategies that enhance, critique and/or extend the role of the news media and its connection to the social. Political scientist Robert Putnam used an analogy at the turn of the century to explain the role of social capital in society. He defined social capital as connections among individuals, networks and the norms of reciprocity and trustworthiness that arises from them, and argued such a resource served like a social glue to hold societies together and a form of social WD40 that lubricated connections and networks (Putnam, 2000). Researchers adopting a Putnam approach have highlighted the role of local newspapers especially as providing the information that connects people, generating social capital or ‘sense of community’. We have adopted social capital theory more broadly to focus on the role and power of local news media to connect people with each other, a practice that builds a local journalist and newspaper’s legitimacy while also enhancing community social capital, identity and progress (Hess, 2014). Ideas around membership building, facilitating community conversations to inform and generate meaningful social connections, solutions and advocacy journalism all fit within this framework because it focuses on the brokerage role journalists play in connecting everyday people with each other, or facilitating connections with those in positions of power. James Carey’s ritualistic view of communication (Carey, 1977) also offers important wisdom to journalism academics that ‘the subject matter of journalism is the conversation the public is having with itself’. Those conversations can be public and social, but what is more important is how local media develop strategies to reclaim their centrality to such conversations by ensuring and creating a constructive space for them to occur in both physical and digital settings. In other words, being able to connect people socially – through the ability to generate a sense of community, bring people together and link them to those in positions of power in the interests of change and progress are powerful tools for news media. Local news outlets which outsource the ‘social’ dimensions of news to Facebook, in our view play a very dangerous game (Hess and Gutsche, 2019).

### The need for coordinated policy/government responses

The power of collective, coordinated responses to COVID-19–from the healthcare sector through to politics, neighbourhoods and schools–has been celebrated across Australia. The pandemic has led to dramatic overhauls of decision-making structures at the highest levels. On May 29, 2020, Prime Minister Scott Morrison announced that the Council of Australian Government (COAG) would cease (https://www.coag.gov.au). As the peak intergovernmental forum established in 1992, its role was to manage matters of national significance, from climate policy to healthcare and education. COAG membership included the Prime Minister, state and territory first ministers and Australian Local Government Association president. It attracted criticism nonetheless because it met irregularly and was considered too bureaucratic. Officials and commentators have used the analogy of the cemetery to describe COAG as a ‘graveyard of good proposals for reform’ (Pelly, 2018); or as the PM stated in April, a place where ‘good ideas went to die’ (Prasser, 2020). Instead a COVID-19 legacy will be the creation of a National Cabinet – devised to develop timely, more effective and collaborative decision making involving the PM, premiers and territory leaders.

It is not our intention to endorse or criticise this response, but it does highlight the need for a reassessment and realignment of existing power structures and the value of a more coordinated response to sustaining local journalism. There are a range of initiatives, administrative bodies, policy think tanks, philanthropic grants, industry and academic research on local news all being circulated without regular collaboration or coordination between key stakeholders. As we highlighted earlier, the Federal Government is administering a $50 million Public Interest Journalism Fund, while the Australian Communication and Media Authority is responsible for a $5 million scheme to support regional publishers. The Judith Neilson Institute is allocating millions of dollars to support journalism projects, as the ACCC is developing a bargaining code to tackle the social media giants (Judith Nielson, 2020).

The dismantling of COAG also resonates with another key challenge in debates about the future of news–the most powerful voices tend to dominate discussion. The Local Government Association, for example, lost its seat at the table of the National Cabinet, prompting concern that rural and regional voices would be lost in future decision making discussions (Vogler, 2020). In media policy debates, there too is concern that News Corp, ACM and Nine will drown out the voices of small town proprietors and community newspapers. Scholars elsewhere have suggested that COVID-19 highlights the important need for a new collaborative funding model to support local news – one that draws on a mix of subscription fees, social media subsidies, advertising revenue and government support to sustain news (see especially Olsen et al., 2020). Such an approach is a useful one, providing we do not overlook issues of power in these debates and that there are mechanisms to ensure those who receive such a mix of support are held accountable to local audiences – in other words, the need for someone to watch the watchdogs (Finkelstein & Ricketson, 2012)

There too remains a serious structural imbalance and lack of coordination in the way different states and territories support local journalism beyond the metropole through government advertising revenue. Victoria is arguably the most committed state, ensuring departments and agencies spend at least 15% of their campaign advertising expenditure on rural and regional media (Victorian Government, 2020). During the pandemic the Premier, Daniel Andrews, also announced a $4.5 million advertising support package for struggling country papers (Andrews, 2020). However, as recently as 2019 South Australia announced legislation that provided freedom for states and local governments to publish information on their own websites, removing their requirement to report public notices in local newspapers, (Government of South Australia, 2020).

### ‘We’re all in this together’–the balance between social order and self interest

From ’we’re all in this together’ posters on shop doors and restaurant windows (Pelly, 2020) rainbow signs on front fences and balconies, to teddy bears in windows, ordinary citizens around the world have joined the World Health Organisation and government leaders in reminding each other ‘we are all in this together’. Local expressions of universal solidarity appeared to ‘go viral’ as people took it upon themselves to act on behalf of others in need (*ABC News*, 2020). At an institutional level, COVID-19 has had the same effect, with medical researchers involved in a world-first collaboration to test experimental treatments for COVID-19 (World Health Organization (WHO), 2020) and grocery giants who usually compete ferociously uniting in a taskforce for the common good and to ensure social order (Powell, 2020).

In his work on the concept of solidarity, German philosopher Kurt Bayertz (1999) traces its roots to the Roman law of obligations, defining it as a mutual attachment between individuals and groups that operates on two levels. The first is the ‘factual’ plane of actual common ground between people; the second is the ‘normative’ level of mutual obligations to aid each other, ‘as and when should be necessary’ (Bayertz, 1999: 3). He uses the term ‘universalistic solidarity’ to describe this, suggesting all human beings have a moral duty to work together for the benefit of all. This is what ‘we’re all in this together’ implies. During times of crisis this can be in the interests of preserving social order, rather than the kind of ‘political solidarity’ that is often formed to enact change or challenge the status quo. But as Bayertz (1999) emphasises, ‘universalistic solidarity’ also ignores differences in power, and potential conflict between the needs and values of different groups. It overshadows how the impact of a crisis is not equal among groups in society, a point emphasised in our earlier discussion of ‘essential services’.

Elsewhere we have argued that there are certain individuals/institutions that are expected to perpetuate moral universalism when it matters most and that people turn to journalism – especially at the local level–for this very purpose (Hess, 2017). This approach provides scope to consider how institutions themselves can unite in the interests of and perpetuate what Bayetrz refers to as solidarity while reinforcing their own self-interest.

Australia’s supermarkets found themselves in uncharted waters when panic buying gripped the nation. Coles and Woolworths have described how they were ‘moving fast but flying blind’ individually, with no chapters in their crisis handbooks on how to deal with a global pandemic (Powell, 2020). With the support of the government, they joined forces with Aldi and IGA to form the first ever Supermarket Taskforce to focus on cross-industry collaboration. Powell (2020) explained that while the taskforce was strictly prohibited by the ACCC from discussing issues such as product pricing, the group shared their plans and best practices across issues like in-store cleaning, social distancing and set the same limits on buying certain products. At the height of the panic buying, the big four supermarkets took out full-page advertisements in major newspapers in an attempt to quell the unrest among shoppers. Woolworths chief Brad Banducci explained how the usually fierce rivals decided the order in which the four companies’ logos would be displayed on the ad:

‘Of course there was only one answer: alphabetically’, he laughs. ‘But that move set a really important tonality that we were all in it together’. (in Powell, 2020)

As the crisis deepened, the Supermarket Taskforce faced some fundamental issues that resonate with Bayetrz’s (1998) observations about the challenges of universal solidarity when it comes to social inequality. They had to find a way to ensure two million vulnerable Australians had food and essential provisions if they were unable to leave the house. Their online delivery systems were ill-suited to the task, so they collaborated with Australia Post and logistics business DHL to organise delivery for an $80 box of essentials. They also instigated a shopping hour for the elderly and vulnerable.

There are lessons here for the legacy media companies who have united on what Bayertz (1999) termed ‘the factual plane of actual common ground’ in the fight against the digital techs, for example, in the ACCC bargaining code framework. Their approach remains disjointed and there are concerns that big players like ACM, News Corp and Nine will drown out the interests of smaller players like small independent newspapers (www.vcpa.com.au). While there is of course self-interest at play here, the Supermarket Taskforce provides an example of competitors working together in the interests of not only wider society, but those in most need, which has taken them into the realm of universal solidarity. They have done this by renegotiating their terms of engagement, the policy frameworks they operate within, and by reaching outside their patch to enlist other collaborators who can help deliver essential services to communities in danger of being severely disadvantaged. As we have discussed previously, the pandemic has crystallised the understanding of country newspapers as ‘essential services’ opening the possibility of developing new approaches to cross-industry collaboration.

On another level, some scholars promote a ‘we are all in this together approach’ by advocating for more collaboration between journalism institutions in the production of good quality and investigative journalism involving public broadcasters, not-for profits and mainstream commercial providers (Jenkins and Graves, 2019). There remains issues around who ultimately benefits from the production of news content, nonetheless.

### Thinking in the future tense: a systemic solution

In advancing a local newspaper innovations agenda, an approach inspired by systems thinking resonates with the overarching analogy of the COVID-19 response explored here. Systems thinking conceptualises the material world as a network of inseparable relationships, patterns and contexts. It accounts for how living networks continually create or recreate themselves by transforming or replacing their components – undergoing continual structural changes while preserving their weblike patterns of organisation. As COVID-19 reminds us, the coexistence of stability and change is one of the key characteristics of life. A system thinking approach can therefore be used to challenge the idea that the most urgent problems facing regional newspapers can be understood as a product of the COVID-19 pandemic, or in isolation from other environmental, political, cultural, social, technological and economic concerns. The problems facing local newspapers require a systemic solution. Elsewhere we have outlined a six-dimensional model that complements this approach to examine media innovation–a model that acknowledges and calls for a critical engagement of these various factors (see Hess and Waller, 2020).

Scientists are looking for innovative ways to protect society from a viral pandemic and are drawing on existing and new treatment options. We too seek new ways of protecting the health of local newspapers while appreciating that not all that is new is innovative and there may be tried and true structures and practices that are fundamental to the future of local news. We are optimistic that taking a fresh conceptual approach to media innovation can generate rich empirical findings and inform the rural news sector for the betterment of society

According to James (1997), systems thinking helps us understand that all parts of a business or a process are connected and to focus on interrelatedness. It involves cooperation, pooling or combining ideas and experience, and can improve innovation, efficiency and performance. In the context of regional and rural newspapers, systems thinking calls for deep engagement with readers and non-readers, as well as those working in the industry and related areas including the businesses that advertise. There are often too many disparate and disconnected voices in the debate about the future of local news who should be brought together, encouraged and supported to work together regularly and constructively. As we have witnessed from the collaborative coordinated response from supermarkets during the pandemic, self-interest can work alongside the greater good, but issues of power must always be examined in the process.

One strategy that emerged from the pandemic that merits further investigation is the value of an independent national cabinet-style body to examine the future and ongoing sustainability of local news. Key stakeholders would include the Federal Communications Department, ACCC, ACMA, philanthropic organisations, community newspaper representatives, key industry representatives, the ABC and academic experts on local media. While the Australian Local Government Association lost it place at the table upon the disbandment of COAG, it most certainly warrants a place within a national consultative body on local media, given it plays an integral and symbiotic role in the survival of local news and a healthy mediated democracy at the grassroots level. In a submission to the ACCC bargaining code in June, 2020, we argued for a digital transition fund to support local newspapers. An administrating body would be needed to fairly divide and allocate any advertising revenue shared by Google and Facebook into the future. Like the National Cabinet established in the midst of the pandemic, a national body for local news would help ensure a more streamlined, effective and targeted approach to supporting rural and regional newspapers into the next decade.

## Conclusion: learning from COVID-19

This essay has argued there is value in analogising as a way of generating fresh perspectives in the debate about the future of local news in Australia. Analogies can be a useful way of ordering and explaining complex ideas and thoughts, and are often adopted by the powerful to outline a point of view. We have begun to map an approach to analogising in media and communication research that focuses on its broader sense-making role, ultimately to help to envisage or reimagine what local journalism might be. In doing so, we acknowledge that the ‘imaginative’ needs to be balanced with a critical examination and reflexive approach to those in positions of privilege who utilise analogies for their advantage.

Positioning the future of news against broader societal responses to COVID-19 provides interesting insight. First, we suggest that we must look beyond the normative role of journalism as ‘an essential service’ to more specifically understand the importance of the printed product in non-metropolitan areas due to demographics and a continuing digital divide. ‘We are all in this together’, meanwhile, has been a powerful reminder of the importance of solidarity in times of crisis. The Supermarket Taskforce offers inspiration for a national body whose mission is not only to ensure local newspapers survive, but to serve the news needs of geographically marginalised audiences.

Much of our research over the past decade has focused on everyday people’s media-related practices to explore the reasons they engage with local news–beyond engagement with matters of politics or a public interest. Over time we have understood the power of the social and the benefits and advantages of those who are central to the social realms that connect with our everyday lives. Facebook and Google position themselves as merely hosts in which harmful bacteria can thrive. Within immunobiology for example, only when the innate host’s own defences are bypassed, evaded, or overwhelmed is an induced immune response required. COVID-19 also tells us that if a host becomes overwhelmed with contagious toxins, they must be isolated and treated as part of the problem.

## Notes

1. We are CIs on the Australian Rresearch Council Linkage project “Media Innovation and the Civic Future of Australia’s country press. [LP180100813]

2. We follow De Certeau (1984) here to define strategies as measures enacted by the powerful from a position of advantage whereas tactics are used by those of us in everyday society to manage our way around or through strategies imposed by institutions. We also acknowledge we are always tied to our lived experiences and histories in such processes (see Bourdieu, 1984).
